# An LPV Adaptive Observer for Updating a Map Applied to an MAF Sensor in a Diesel Engine

**DOI:** 10.3390/s151027142

**Published:** 2015-10-23

**Authors:** Zhiyuan Liu, Changhui Wang

**Affiliations:** Department of Control Science and Engineering, Harbin Institute of Technology, Harbin 150001, China; E-Mail: liuzy_hit@hit.edu.cn

**Keywords:** linear parameter varying (LPV) system, adaptive observer, sensor error compensation, map (or lookup table) estimation, mass air flow, diesel engine

## Abstract

In this paper, a new method for mass air flow (MAF) sensor error compensation and an online updating error map (or lookup table) due to installation and aging in a diesel engine is developed. Since the MAF sensor error is dependent on the engine operating point, the error model is represented as a two-dimensional (2D) map with two inputs, fuel mass injection quantity and engine speed. Meanwhile, the 2D map representing the MAF sensor error is described as a piecewise bilinear interpolation model, which can be written as a dot product between the regression vector and parameter vector using a membership function. With the combination of the 2D map regression model and the diesel engine air path system, an LPV adaptive observer with low computational load is designed to estimate states and parameters jointly. The convergence of the proposed algorithm is proven under the conditions of persistent excitation and given inequalities. The observer is validated against the simulation data from engine software enDYNA provided by Tesis. The results demonstrate that the operating point-dependent error of the MAF sensor can be approximated acceptably by the 2D map from the proposed method.

## 1. Introduction

Accurate exhaust gas recirculation (EGR) rate control and air-fuel ratio (AFR) control are important technologies to satisfy the increasingly stringent emission regulations, which are dependent on the precise calculation of the EGR rate and AFR [1,2]. The accuracy of the EGR rate and AFR can be improved by a mass air flow (MAF) sensor, in which a sensor element is heated to a fixed temperature, and the difference in temperature attributed to heat transfer from the heating element to the air flow is a measure of the air mass flow [3,4,5]. However, there are many different local flow fields within the inlet piping due to the three-dimensional turbulence flow, leading to measurement biases in the MAF sensor installed between the air filter and the intake manifold. In addition, the MAF sensor is also subjected to aging phenomena owing to the accumulation of dust on the sensing element, which causes the deterioration of the measurement accuracy [6,7]. These errors will bring about an inaccurate EGR rate and AFR and have adverse impacts on the emission performance of diesel engine.

It is difficult to accurately establish an analytical model for the MAF sensor error. In view of the relatively low computational load, maps (or lookup tables) have been widely used to characterize systems where the functional relationship is unavailable or too complex to represent analytically [8]. Therefore, the relative error of the MAF sensor is described as a one-dimensional (1D) map taking compressor mass air flow as input [2]. In order to track MAF sensor aging, the extended Kalman filter (EKF) for updating maps is presented in [9,10,11], in which the 1D map is represented as a piecewise linear interpolation model and the map parameters are considered as parameter states. Due to the piecewise linear interpolation model having the characteristic of partition calculation and due to the the map input being able to enter only one input interval of the 1D map at any time, then only two parameter states participating in linear interpolation are observable and the other not. Therefore, the error covariance matrix elements of EKF corresponding to the locally unobservable parameter states will increase linearly. Although the solution is to limit this growth in [9,10,11], the convergence of EKF with a confined covariance matrix cannot be guaranteed. In addition, the measurement error of the MAF sensor depends on the engine operating point, which is usually defined as fuel mass injection quantity and engine speed. The 1D map representing MAF sensor error ignores the engine speed, reducing the accuracy when the diesel engine is run over a wide speed range.

The adaptive observer with the advantage of simple convergence conditions is an alternative method for updating maps. Recursive algorithms designed for joint estimation of states and parameters in state space systems are usually known as adaptive observers, and some early works with adaptive observers to jointly estimate states and parameters in multi-input-multi-output linear time varying systems can be found in [12,13]. In order to estimate sensor faults, adaptive observers for linear time varying systems with unknown parameters in output equations have been studied [14,15]. However, the existing adaptive observers cannot directly update maps.

In this paper, an adaptive observer is developed to update the map, in which the MAF sensor error is described as a two-dimensional (2D) map taking the operating point as the input to improve the model accuracy comparing the 1D map. Then, two problems are studied. First, in order to expediently analyze and design the parameter estimation method, the input-output relationship of the MAF sensor error 2D map is expressed as a dot product between the regression vector and the unknown parameter vector. Second, based on the linear parameter varying (LPV) system of the diesel engine with EGR and variable geometry turbocharger (VGT), a 2D map estimation method with a simple structure and low computational load is designed to facilitate the algorithm implementation.

This paper is organized as follows. In Section 2, the 2D map is expressed as the dot product between the regression vector and the unknown parameter vector, and the estimation problem for a class of LPV systems with an unknown parameter vector is given. In Section 3, the LPV adaptive observer is proposed, as well as the convergence analysis. Simulation results from enDYNA are presented in Section 4, and the conclusions are summarized in Section 5.

## 2. Problem Formulation

### 2.1. A Diesel Engine Air Path LPV Model

**Figure 1:**
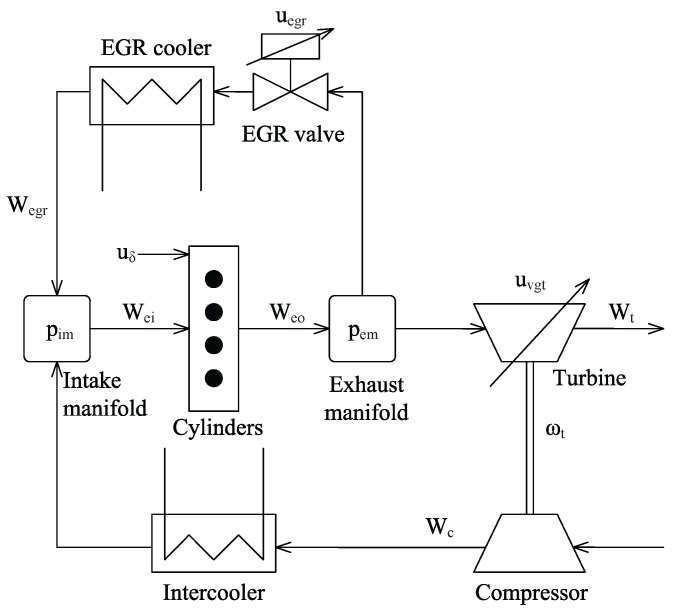
Schematic of the diesel engine model with exhaust gas recirculation (EGR) and variable geometry turbocharger (VGT).

Figure 1 shows the model structure of a diesel engine with EGR and VGT, and the model can be expressed as [16]:

(1) $$\begin{array}{cc} {\dot{p}}_{im} & =\frac{R_{a}T_{im}}{V_{im}}\left(W_{c}+W_{egr}-W_{ei}\right)\\ {\dot{p}}_{em} & =\frac{R_{e}T_{em}}{V_{em}}\left(W_{f}+W_{ei}-W_{t}-W_{egr}\right)\\ {\dot{\omega }}_{t} & =\frac{P_{t}\eta_{m}-P_{c}}{J_{t}\omega_{t}} \end{array}$$

where $W_{c}$ is the compressor mass air flow, $W_{egr}$ is the EGR mass flow, $W_{ei}$ is the cylinder mass flow, $W_{f}$ is the fuel rate injected to cylinder, $W_{t}$ is the turbine mass flow, $P_{t}$ is the turbine power, $P_{c}$ is the compressor power, $\eta_{m}$ is the turbocharger mechanical efficiency, $p_{im}$ is the intake manifold pressure, $p_{em}$ is the exhaust manifold pressure and $\omega_{t}$ is the turbine speed.

Meanwhile, $W_{c}$, $W_{egr}$, $W_{ei}$, $W_{t}$, $W_{f}$, $P_{c}$ and $P_{t}\eta_{m}$ in Equation (1) can be obtained as follows:

(2) Wc=pambπRc3·Φcpim,ωtRaTambωt,Wegr=Aegruegr·Ψegrpim,pemTemRepemWei=ηvolpim,ne·neVd120RaTimpim,Wt=Avgtmax·fΠtpem·fvgtuvgtTemRepemWf=10-6120ncylneuδ,Pc=Wcpim,ωt·cpaTamb·Πc1-11γaγa-1ηcpim,ωtPtηm=ηtmpem,ωt·Wtpem,uvgt·cpeTem·1-Πt1-11γeγe

However, it is difficult to estimate the measurement error of the MAF sensor based on the complicated nonlinear model Equation (1). In order to simply present the state space equation and the error estimation, define variables:

(3) ρ1=pambπRc3·Φcpim,ωtRaTamb,ρ2=Aegruegr·Ψegrpim,pemTemReρ3=ηvolpim,ne·neVd120RaTim,ρ4=Avgtmax·fΠtpem·fvgtuvgtTemReρ5=cpapambπRc3·Φcpim,ωt·Πc1-11γaγa-1JtωtRa·ηcpim,ωt

According to Equation (3), the variables $\rho_{i}\;\left(i=1,2,3,4,5\right)$ are available in real-time since $p_{im}$, $p_{em}$, $\omega_{t}$, $u_{egr}$, $u_{vgt}$, $u_{\delta }$ and $n_{e}$ can be measured or estimated online. Therefore, the nonlinear model Equation (1) can be cast into an LPV system:

(4) $$\begin{array}{c} \dot{x}=A\left(\rho \right)x+E \end{array}$$

where:

(5) ρ=ρ1ρ2ρ3ρ4ρ5x=pimpemωt,Aρ=−a1ρ3a1ρ2a1ρ1a2ρ3−a2ρ2−a2ρ4000−ρ5E=0a2WfPtηmPtηmJtωtJtωt,a1=RaTimVim,a2=ReTemVem

In order to determine the bounds on the parameter vector *ρ*, a simulation study is performed using a 1.9 L four-cylinder common rail turbo diesel engine of enDYNA provided by Tesis [17,18]. The bounds of the parameter vector *ρ* are found using the simulation data from enDYNA over the European Transient Cycle (ETC), Federal Test Procedure 75 (FTP75) and New European Drive Cycle (NEDC) [19,20,21]. Then, the results are listed in Table 1. It follows that each parameter $\rho_{i}$ from parameter vector *ρ* is bounded by a minimum and maximum value ${\underline{\rho }}_{i}$ and ${\bar{\rho }}_{i}$.

**Table 1:** Bounds on the parameter vector *ρ* under three conditions. ETC, European Transient Cycle; FTP75, Federal Test Procedure 75; NEDC, New European Drive Cycle.

Parameter	ETC	FTP75	NEDC
$\rho_{1}$	$\left[19,150\right]\times 10^{-7}$	$\left[146,110\right]\times 10^{-7}$	$\left[49,130\right]\times 10^{-7}$
$\rho_{2}$	$\left[-1.65,1.6\right]\times 10^{-7}$	$\left[-0.56,1.15\right]\times 10^{-7}$	$\left[-0.003,1.0\right]\times 10^{-7}$
$\rho_{3}$	$\left[1.09,6.4\right]\times 10^{-7}$	$\left[1.18,6\right]\times 10^{-7}$	$\left[1.1,5.4\right]\times 10^{-7}$
$\rho_{4}$	$\left[0.61,6.01\right]\times 10^{-7}$	$\left[0.75,5.4\right]\times 10^{-7}$	$\left[0.62,4.7\right]\times 10^{-7}$
$\rho_{5}$	[0,3.15]	[0,2.78]	[0,2.67]

### 2.2. 2D Map Description for the MAF Sensor Error

The intake manifold pressure $p_{im}$, turbine speed $\omega_{t}$ and compressor mass air flow $W_{c}$ are the outputs of interest to analyze the MAF sensor error, which is:

(6) $$\begin{array}{c} y=\left(\begin{array}{c} y_{1}\\ y_{2} \end{array}\right)=\left(\begin{array}{c} p_{im}\\ \omega_{t}\\ W_{c} \end{array}\right) \end{array}$$

where $y_{1}={\left(\begin{array}{cc} p_{im} & \omega_{t} \end{array}\right)}^{T},y_{2}=W_{c}$. Due to the existence of MAF sensor error, the output Equation (6) becomes:

(7) $$\begin{array}{c} y_{m}=\left(\begin{array}{c} y_{1m}\\ y_{2m} \end{array}\right)=\left(\begin{array}{c} y_{1}\\ y_{2}+\Delta W_{c} \end{array}\right) \end{array}$$

where $y_{m}$ is the measured value from sensors. ${\Delta W}_{c}$ is the measurement error of the MAF sensor, which depends on the engine operating point (fuel mass injection quantity $u_{\delta }$ and engine speed $n_{e}$), *i.e.*, $\Delta W_{c}\left(u_{\delta }.n_{e}\right)$. Since it is difficult to accurately build an analytical model for $\Delta W_{c}\left(u_{\delta }.n_{e}\right)$, a 2D map is adopted in this paper to describe $\Delta W_{c}\left(u_{\delta },n_{e}\right)$. Therefore, define the partition of the 2D map input $\upsilon =\left(u_{\delta },n_{e}\right)$ as:

(8) $$\begin{array}{c} a=u_{\delta }^{1}<u_{\delta }^{2}<\ldots <u_{\delta }^{p_{1}}=b\\ c=n_{e}^{1}<n_{e}^{2}<\ldots <n_{e}^{p_{2}}=d \end{array}$$

where a,b∈R are the minimum and maximum values of $u_{\delta }$ and $p_{1}$ is the number of the grid points in $\left[a,b\right]$. c,d∈R are the minimum and maximum values of $n_{e}$, and $p_{2}$ is the number of the grid points in $\left[c,d\right]$.

Assume that the measurement error of the input grid points $\left(u_{\delta }^{i},n_{e}^{j}\right)$ is $\theta^{i,j}$, *i.e.*,

(9) $$\begin{array}{c} \theta^{i,j}=\Delta W_{c}\left(u_{\delta }^{i},n_{e}^{j}\right)\\ i=1,2,\cdots p_{1};j=1,2,\cdots p_{2} \end{array}$$

Then, for $\forall \upsilon \in \left[u_{\delta }^{i},u_{\delta }^{i+1}\right]\times \left[n_{e}^{j},n_{e}^{j+1}\right]$, $\forall i\in \left[1,2,\cdots ,p_{1}-1\right]$ and $\forall j\in \left[1,2,\cdots ,p_{2}-1\right]$, we can hold the $n_{e}$ value fixed and apply one dimensional (1D) linear interpolation in the $u_{\delta }$ direction. Using the Lagrange form, the result is:

(10) $$\begin{array}{c} q_{u_{\delta }}^{j}\left(\theta^{i,j}\right)=\frac{u_{\delta }^{i+1}-u_{\delta }}{u_{\delta }^{i+1}-u_{\delta }^{i}}\theta^{i,j}+\frac{u_{\delta }-u_{\delta }^{i}}{u_{\delta }^{i+1}-u_{\delta }^{i}}\theta^{i+1,j}\\ u_{\delta }\in \left[u_{\delta }^{i},u_{\delta }^{i+1}\right],i=1,2,\cdots p_{1}-1 \end{array}$$

Equation (10) can then be used to linearly interpolate along the $n_{e}$ dimension to yield the piecewise bilinear interpolation model of the measurement error $\Delta W_{c,T}\left(\theta^{i,j},\upsilon \right)$ as:

(11) $$\begin{array}{c} \Delta W_{c,T}\left(\theta^{i,j},\upsilon \right)=\frac{n_{e}^{j+1}-n_{e}}{n_{e}^{j+1}-n_{e}^{j}}q_{u_{\delta }}^{j}\left(\theta^{i,j}\right)+\frac{n_{e}-n_{e}^{j}}{n_{e}^{j+1}-n_{e}^{j}}q_{u_{\delta }}^{j+1}\left(\theta^{i,j}\right)\\ \upsilon \in \left[u_{\delta }^{i},u_{\delta }^{i+1}\right]\times \left[n_{e}^{j},n_{e}^{j+1}\right],i=1,\cdots p_{1}-1;j=1,\cdots p_{2}-1 \end{array}$$

For the undefined region $\upsilon \in \left(R\times R\right)\backslash \left(\left[a,b\right]\times \left[c,d\right]\right)$, we extend Equation (11) to the final result:

(12) $$\Delta W_{c,T}\left(\theta^{i,j},\upsilon \right)=\left\{\begin{array}{cc} q_{u_{\delta }}^{1}\left(\theta^{i,j}\right) & ,u\in R\times R_{n_{e}}^{0}\\ \frac{n_{e}^{j\;+\;1}-n_{e}}{n_{e}^{j\;+\;1}-n_{e}^{j}}q_{u_{\delta }}^{j}\left(\theta^{i,j}\right)+\frac{n_{e}-n_{e}^{j}}{n_{e}^{j\;+\;1}-n_{e}^{j}}q_{u_{\delta }}^{j\;+\;1}\left(\theta^{i,j}\right) & ,u\in R\times R_{n_{e}}^{j}\\ q_{u_{\delta }}^{p_{2}}\left(\theta^{i,j}\right) & ,u\in R\times R_{n_{e}}^{p_{2}} \end{array}\right.$$

where:

(13) $$\begin{array}{c} q_{u_{\delta }}^{j}\left(\theta^{i,j}\right)=\left\{\begin{array}{cc} \theta^{1,j} & ,u_{\delta }\in R_{u_{\delta }}^{0}\\ \frac{u_{\delta }^{i+1}-u_{\delta }}{u_{\delta }^{i+1}-u_{\delta }^{i}}\theta^{i,j}+\frac{u_{\delta }-u_{\delta }^{i}}{u_{\delta }^{i+1}-u_{\delta }^{i}}\theta^{i+1,j} & ,u_{\delta }\in R_{u_{\delta }}^{i}\\ \theta^{p_{1},j} & ,u_{\delta }\in R_{u_{\delta }}^{p_{1}} \end{array}\right.\\ i=1,2,\cdots p_{1}-1;j=1,2,\cdots p_{2}-1 \end{array}$$

and:

(14) $$R_{u_{\delta }}^{k}=\left\{\begin{array}{cc} \left(-\infty ,u_{\delta }^{1}\right], & k\;=\;0\\ \left(u_{\delta }^{k},u_{\delta }^{k+1}\right], & k\;=\;1,\ldots ,p_{1}-1\\ \left(u_{\delta }^{p_{1}},+\infty \right), & k\;=\;p_{1} \end{array}\right.$$

(15) $$R_{n_{e}}^{l}=\left\{\begin{array}{cc} \left(-\infty ,n_{e}^{1}\right], & l\;=\;0\\ \left(n_{e}^{l},n_{e}^{l+1}\right], & l\;=\;1,\ldots ,p_{2}-1\\ \left(n_{e}^{p_{2}},+\infty \right), & l\;=\;p_{2} \end{array}\right.$$

For the purposes of estimating unknown parameter $\theta^{i,j}$ in $\Delta W_{c,T}\left(\theta^{i,j},\upsilon \right)$ expediently, Equation (12) in vector-vector form is needed. According to the input interval Equations (14) and (15), we define membership function as:

(16) $$\begin{array}{c} \delta_{u_{\delta }}^{k}=\left\{\begin{array}{cc} 1, & u_{\delta }\in R_{u_{\delta }}^{k}\\ 0, & other \end{array}\right.\\ k=0,1,\cdots ,p_{1} \end{array}$$

and:

(17) $$\begin{array}{c} \delta_{n_{e}}^{l}=\left\{\begin{array}{cc} 1, & n_{e}\in R_{n_{e}}^{l}\\ 0, & other \end{array}\right.\\ l\;=\;0,1,\ldots ,p_{2} \end{array}$$

Using membership function Equations (16) and (17), Equation (12) becomes:

(18) $$\begin{array}{cc} \Delta W_{c,T}\left(\theta^{i,j},\upsilon \right) & =\delta_{n_{e}}^{0}q_{u_{\delta }}^{1}\left(\theta^{i,j}\right)+\sum_{j=1}^{p_{2}-1}\delta_{n_{e}}^{j}\left(\frac{n_{e}^{j+1}-n_{e}}{n_{e}^{j+1}-n_{e}^{j}}q_{u_{\delta }}^{j}\left(\theta^{i,j}\right)+\frac{n_{e}-n_{e}^{j}}{n_{e}^{j+1}-n_{e}^{j}}q_{u_{\delta }}^{j+1}\left(\theta^{i,j}\right)\right)+\delta_{n_{e}}^{p_{2}}q_{u_{\delta }}^{p_{2}}\left(\theta^{i,j}\right)\\ & =\Psi_{n_{e}}\cdot q_{u_{\delta }}\left(\theta^{i,j}\right) \end{array}$$

where:

(19) $$\begin{array}{c} \Psi_{n_{e}}={\left(\begin{array}{c} \delta_{n_{e}}^{0}+\delta_{n_{e}}^{1}\frac{n_{e}^{2}-n_{e}}{n_{e}^{2}-n_{e}^{1}}\\ \delta_{n_{e}}^{1}\frac{n_{e}-n_{e}^{1}}{n_{e}^{2}-n_{e}^{1}}+\delta_{n_{e}}^{2}\frac{n_{e}^{3}-n_{e}}{n_{e}^{3}-n_{e}^{2}}\\ \vdots \\ \delta_{n_{e}}^{p_{1}-2}\frac{n_{e}-n_{e}^{p_{2}-2}}{n_{e}^{p_{2}-1}-n_{e}^{p_{2}-2}}+\delta_{n_{e}}^{p_{1}-1}\frac{n_{e}^{p_{2}}-n_{e}}{n_{e}^{p_{2}}-n_{e}^{p_{2}-1}}\\ \delta_{n_{e}}^{p_{1}-1}\frac{n_{e}-n_{e}^{p_{2}-1}}{n_{e}^{p_{2}}-n_{e}^{p_{2}-1}}+\delta_{n_{e}}^{p_{1}} \end{array}\right)}^{T},q_{u_{\delta }}\left(\theta^{i,j}\right)=\left(\begin{array}{c} q_{u_{\delta }}^{1}\left(\theta^{i,j}\right)\\ q_{u_{\delta }}^{2}\left(\theta^{i,j}\right)\\ \vdots \\ q_{u_{\delta }}^{p_{2}-1}\left(\theta^{i,j}\right)\\ q_{u_{\delta }}^{p_{2}}\left(\theta^{i,j}\right) \end{array}\right) \end{array}$$

and:

(20) $$\begin{array}{cc} q_{u_{\delta }}^{j}\left(\theta^{i,j}\right) & =\delta_{u_{\delta }}^{0}\theta^{1,j}+\sum_{i=1}^{p_{1}-1}\delta_{u_{\delta }}^{i}\left(\frac{u_{\delta }^{i+1}-u_{\delta }}{u_{\delta }^{i+1}-u_{\delta }^{i}}\theta^{i,j}+\frac{u_{\delta }-u_{\delta }^{i}}{u_{\delta }^{i+1}-u_{\delta }^{i}}\theta^{i+1,j}\right)+\delta_{u_{\delta }}^{p_{1}}\theta^{p_{1},j}\\ & =\Psi_{u_{\delta }}\cdot \theta_{u_{\delta }}^{j} \end{array}$$

where:

(21) $$\begin{array}{c} \Psi_{u_{\delta }}={\left(\begin{array}{c} \delta_{u_{\delta }}^{0}+\delta_{u_{\delta }}^{1}\frac{u_{\delta }^{2}-u_{\delta }}{u_{\delta }^{2}-u_{\delta }^{1}}\\ \delta_{u_{\delta }}^{1}\frac{u_{\delta }-u_{\delta }^{1}}{u_{\delta }^{2}-u_{\delta }^{1}}+\delta_{u_{\delta }}^{2}\frac{u_{\delta }^{3}-u_{\delta }}{u_{\delta }^{3}-u_{\delta }^{2}}\\ \vdots \\ \delta_{u_{\delta }}^{p_{1}-2}\frac{u_{\delta }-u_{\delta }^{p_{1}-2}}{u_{\delta }^{p_{1}-1}-u_{\delta }^{p_{1}-2}}+\delta_{u_{\delta }}^{p_{1}-1}\frac{u_{\delta }^{p_{1}}-u_{\delta }}{u_{\delta }^{p_{1}}-u_{\delta }^{p_{1}-1}}\\ \delta_{u_{\delta }}^{p_{1}-1}\frac{u_{\delta }-u_{{}_{\delta }}^{p_{1}-1}}{u_{\delta }^{p_{1}}-u_{\delta }^{p_{1}-1}}+\delta_{u_{\delta }}^{p_{1}} \end{array}\right)}^{T},\theta_{u_{{}_{\delta }}}^{j}=\left(\begin{array}{c} \theta^{1,j}\\ \theta^{2,j}\\ \vdots \\ \theta^{p_{1}-1,j}\\ \theta^{p_{1},j} \end{array}\right) \end{array}$$

and $\Psi_{n_{e}}\in R^{1\times p_{1}},\Psi_{n_{e}}\in R^{1\times p_{2}},\theta_{u_{{}_{\delta }}}^{j}\in R^{p_{1}\times 1},q_{u_{\delta }}\left(\theta^{i,j}\right)\in R^{p_{2}\times 1}$.

Now, following Equations (18)–(21), $\Delta W_{c,T}\left(\theta^{i,j},\upsilon \right)$ can be written as a dot product between regression vector $\Psi \left(\upsilon \right)$ and unknown parameter vector *θ* as follows:

(22) $$\begin{array}{c} \Delta W_{c,T}\left(\theta ,\upsilon \right)=\Psi \left(\upsilon \right)\cdot \theta ,\forall \upsilon \in R\times R \end{array}$$

where:

(23) $$\begin{array}{c} \Psi \left(\upsilon \right)=\Psi_{n_{e}}\cdot \left(\begin{array}{cccc} \Psi_{u_{\delta }} & 0 & \cdots & 0\\ 0 & \Psi_{u_{\delta }} & \cdots & 0\\ \vdots & \vdots & \ddots & \vdots \\ 0 & 0 & \cdots & \Psi_{u_{\delta }} \end{array}\right),\theta =\left(\begin{array}{c} \theta_{u_{\delta }}^{1}\\ \theta_{u_{\delta }}^{2}\\ \vdots \\ \theta_{u_{\delta }}^{p_{2}} \end{array}\right) \end{array}$$

and $\Psi \left(\upsilon \right)\in R^{1\times p},\theta \in R^{p\times 1},p=p_{1}\cdot p_{2}$.

With the combination of Equations (4), (7) and (22), the diesel engine air path LPV model can be described by the following state space equation:

(24) $$\begin{array}{cc} \dot{x} & =A\left(\rho \right)x+E\\ y_{m} & =C\left(\rho \right)x+G\Psi \left(\upsilon \right)\theta \end{array}$$

where:

(25) $$\begin{array}{c} C\left(\rho \right)=\left(\begin{array}{ccc} 1 & 0 & 0\\ 0 & 0 & 1\\ 0 & 0 & \rho_{1} \end{array}\right),G=\left(\begin{array}{c} 0\\ 0\\ 1 \end{array}\right) \end{array}$$

Equation (24) indicates that the estimation of the MAF sensor error $\Delta W_{c}\left(u_{\delta },n_{e}\right)$ becomes joint estimation of state *x* and parameter *θ* for LPV system Equation (24).

## 3. Adaptive Observer Design

The observer to estimate state *x* and parameter *θ* jointly for the LPV system Equation (24) is given:

(26) $$\begin{array}{cc} \dot{\hat{x}} & =A\left(\rho \right)\hat{x}+E+L\left(y_{m}-C\left(\rho \right)\hat{x}-G\Psi \left(\upsilon \right)\hat{\theta }\right)\\ \dot{\hat{\theta }} & =\Gamma \Psi {\left(\upsilon \right)}^{T}\left(y_{m2}-C_{2}\left(\rho \right)\hat{x}-\Psi \left(\upsilon \right)\hat{\theta }\right) \end{array}$$

where $C_{2}\left(\rho \right)=\left(\begin{array}{ccc} 0 & 0 & \rho_{1} \end{array}\right)$, $\hat{x}\in R^{3\times 1}$ is the state estimate, $\hat{\theta }\in R^{p\times 1}$ is the parameter estimate, gain $\Gamma \in R^{p\times p}$ is the positive definite diagonal matrix and $L\in R^{3\times 3}$ is the feedback gain matrix.

The asymptotical stability of the proposed algorithm Equation (26) is analyzed in the following theorem.

**Theorem 1.**  *If the following Conditions (1) and (2) hold, then LPV adaptive observer Equation (26) is asymptotically stable, i.e., for any initial conditions* $x\left(0\right),\hat{x}\left(0\right),\hat{\theta }\left(0\right)$ *and parameter vector θ, the errors $\hat{x}-x$ and $\hat{\theta }-\theta$ tend to zero asymptotically when t→∞*.

*(1) There exist matrices L, $P=P^{T}>0$, $Q=Q^{T}>0$ and constant $\varepsilon_{1},\varepsilon_{2}>0$, such that the following set of linear matrix inequalities (LMIs) is feasible for $\forall \rho_{i}\in \left[{\underline{\rho }}_{i},{\bar{\rho }}_{i}\right]$, i=1,2,3,4,5:*

(27) $$M\left(\rho \right)=\left[\begin{array}{ccc} A_{cl}{\left(\rho \right)}^{T}P+PA_{cl}\left(\rho \right)+Q & PLG & C_{2}^{T}\left(\rho \right)\\ G^{T}L^{T}P^{T} & -\varepsilon_{1}I & 0\\ C_{2}\left(\rho \right) & 0 & -\varepsilon_{2}I \end{array}\right]<0$$

(28) $$\begin{array}{c} 2-\varepsilon_{1}-\varepsilon_{2}>0 \end{array}$$

*where $A_{cl}\left(\rho \right)=A\left(\rho \right)-LC\left(\rho \right)$* .

*(2) There exists map input υ, such that the regression vector $\Psi \left(\upsilon \right)$ is persistently exciting, i.e., $\exists \delta_{1},\delta_{2}>0;\exists T>0;\forall t\geq 0$:*

(29) $$\begin{array}{c} \delta_{1}I_{p}\leq \int_{t}^{t+T}\Psi {\left(\upsilon \left(\tau \right)\right)}^{T}\Psi \left(\upsilon \left(\tau \right)\right)d\tau \leq \delta_{2}I_{p} \end{array}$$

**Proof.**  Set the estimation error $\tilde{x}=\hat{x}-x,\tilde{\theta }=\hat{\theta }-\theta$. Notice that $\dot{\theta }=0$; the error dynamic system between Equations (24) and (26) is:

(30) $$\begin{array}{cc} \dot{\tilde{x}} & =\left(A\left(\rho \right)-LC\left(\rho \right)\right)\tilde{x}-LG\Psi \left(\upsilon \right)\tilde{\theta }\\ \dot{\tilde{\theta }} & =-\Gamma \Psi {\left(\upsilon \right)}^{T}\left(C_{2}\left(\rho \right)\tilde{x}+\Psi \left(\upsilon \right)\tilde{\theta }\right) \end{array}$$

A valid Lyapunov function candidate is considered as $V=\eta^{T}P\eta +{\tilde{\theta }}^{T}\Gamma^{-1}\tilde{\theta }$. For $\forall \tilde{x}\neq 0$, the derivative of *V* along with the error dynamic system Equation (30) is:

(31) $$\begin{array}{cc} \dot{V} & =2{\tilde{x}}^{T}P\dot{\tilde{x}}+2{\tilde{\theta }}^{T}\Gamma^{-1}\dot{\tilde{\theta }}\\ & =2{\tilde{x}}^{T}PA_{cl}\left(\rho \right)\tilde{x}-2{\tilde{\theta }}^{T}\Psi {\left(\upsilon \right)}^{T}\Psi \left(\upsilon \right)\tilde{\theta }-2{\tilde{x}}^{T}PLG\Psi \left(\upsilon \right)\tilde{\theta }-2{\tilde{\theta }}^{T}\Psi {\left(\upsilon \right)}^{T}C_{2}\left(\rho \right)\tilde{x} \end{array}$$

There exist $\varepsilon_{1},\varepsilon_{2}>0$, such that the following inequalities hold:

$$\begin{array}{c} -2{\tilde{x}}^{T}PLG\Psi \left(\upsilon \right)\tilde{\theta }⩽{∥\frac{1}{\sqrt{\varepsilon_{1}}}{\tilde{x}}^{T}PLG∥}^{2}+{∥\sqrt{\varepsilon_{1}}\Psi \left(\upsilon \right)\tilde{\theta }∥}^{2}\\ -2{\tilde{\theta }}^{T}\Psi {\left(\upsilon \right)}^{T}C_{2}\left(\rho \right)\tilde{x}⩽{∥\frac{1}{\sqrt{\varepsilon_{2}}}C_{2}\left(\rho \right)\tilde{x}∥}^{2}+{∥\sqrt{\varepsilon_{2}}{\tilde{\theta }}^{T}\Psi {\left(\upsilon \right)}^{T}∥}^{2} \end{array}$$

According to Condition (1) and Equation (31), the following inequality holds:

$$\begin{array}{cc} \dot{V} & \leq {\tilde{x}}^{T}\left(A_{cl}{\left(\rho \right)}^{T}P+PA_{cl}\left(\rho \right)+\frac{1}{\varepsilon_{1}}PLGG^{T}L^{T}P+\frac{1}{\varepsilon_{2}}C_{2}^{T}\left(\rho \right)C_{2}\left(\rho \right)\right)\tilde{x}\\ & -\left(2-\varepsilon_{1}-\varepsilon_{2}\right){\tilde{\theta }}^{T}\Psi {\left(\upsilon \right)}^{T}\Psi \left(\upsilon \right)\tilde{\theta }\\ & <-{\tilde{x}}^{T}Q\tilde{x}<0 \end{array}$$

That is $\dot{V}<-\omega \left(t\right)<0$, for $\forall \rho_{i}\in \left[{\underline{\rho }}_{i},{\bar{\rho }}_{i}\right],i=1,2,3,4,5$, where $\omega \left(t\right)={\tilde{x}}^{T}Q\tilde{x}$. Based on the Lyapunov stability theory, we know that the equilibrium $\tilde{x}=0$ and $\tilde{\theta }=0$ are stable. Now, integrating $\dot{V}<-\omega \left(t\right)$ from zero to *t* yields:

(32) $$\begin{array}{c} V\left(t\right)+\int_{0}^{t}\omega \left(\tau \right)d\tau <V\left(0\right) \end{array}$$

and this means that $\int_{0}^{t}\omega \left(\tau \right)d\tau <V\left(0\right)$ since V>0. Therefore, we have $\lim_{t\to \infty }\int_{0}^{t}\omega \left(\tau \right)d\tau \leq V\left(0\right)$, and this implies that $\lim_{t\to \infty }\int_{0}^{t}\omega \left(\tau \right)d\tau$ exists and is finite. By Barbalat’s Lemma [22], we know that $\lim_{t\to \infty }\omega \left(t\right)=0$, and this leads to $\lim_{t\to \infty }\tilde{x}\left(t\right)=0$.

Under Condition (2), the vector $\Psi \left(\upsilon \right)$ is persistently exciting, that is we have $\lim_{t\to \infty }\tilde{\theta }\left(t\right)=0$ [22]. ☐

**Remark 1.**  *With the concept of multi-convexity [23], the solution of the infinite LMI Equation (27) can be reduced to be a solution of the finite LMIs for the vertex set, that is:*

(33) $$\begin{array}{c} M\left(w\right)<0,\forall w\in V=\left\{\left.\left(w_{1},\cdots ,w_{5}\right)\right|w_{i}\in \left\{{\underline{\rho }}_{i},{\bar{\rho }}_{i}\right\},i=1,2,3,4,5\right\} \end{array}$$

*Therefore, feedback gain L can be obtained by the solution of inequality Equations (28) and (33)*.

**Remark 2.**  *With the membership function $\delta_{u_{\delta }}^{k},\delta_{n_{e}}^{l}$ in Equations (16) and (17), we know that $\delta_{u_{\delta }}^{k}$ = $\delta_{n_{e}}^{l}$ = 1 when $\upsilon \in R_{u_{\delta }}^{k}\times R_{n_{e}}^{l}$ and $\delta_{u_{\delta }}^{k}\;=\;\delta_{n_{e}}^{l}\;=\;0$ when $\upsilon \notin R_{u_{\delta }}^{k}\times R_{n_{e}}^{l}$. Therefore, the regression vector $\Psi \left(\upsilon \right)$ is a sparse vector*.

According to the partition of the map input $\upsilon =\left(u_{\delta },n_{e}\right)$ defined in Equation (8) and the piecewise bilinear interpolation model Equation (12), the input *υ* (engine operating point) moves in only one region $R_{u_{\delta }}^{k}\times R_{n_{e}}^{l}$ at any time, and only the parameters ${\hat{\theta }}^{i,j}$ corresponding to the region $R_{u_{\delta }}^{k}\times R_{n_{e}}^{l}$ can participate in the interpolation. That is, for $\forall \upsilon \in R_{u_{\delta }}^{k}\times R_{n_{e}}^{l}$:

Case 1: $\left(k,l\right)\in \left\{0,p_{1}\right\}\times \left\{0,p_{2}\right\}$. Only one parameter ${\hat{\theta }}^{i,j},\left(i,j\right)\in \left\{1,p_{1}\right\}\times \left\{1,p_{2}\right\}$ takes part in the interpolation, *i.e.*, $\Delta W_{c,T}\left({\hat{\theta }}^{i,j},\upsilon \right)={\hat{\theta }}^{i,j}$.

Case 2: $\left(k,l\right)\in \left\{1,2,\cdots p_{1}-1\right\}\times \left\{0,p_{2}\right\}$. Two parameters ${\hat{\theta }}^{i,j},{\hat{\theta }}^{i+1,j},\left(i,j\right)\in \left\{1,2,\cdots p_{1}-1\right\}\times \left\{1,p_{2}\right\}$ take part in the interpolation, *i.e.*,

$$\Delta W_{c,T}\left({\hat{\theta }}^{i,j},\upsilon \right)=\frac{u_{\delta }^{i+1}-u_{\delta }}{u_{\delta }^{i+1}-u_{\delta }^{i}}{\hat{\theta }}^{i,j}+\frac{u_{\delta }-u_{\delta }^{i}}{u_{\delta }^{i+1}-u_{\delta }^{i}}{\hat{\theta }}^{i+1,j}$$

Case 3: $\left(k,l\right)\in \left\{0,p_{1}\right\}\times \left\{1,2,\cdots p_{2}-1\right\}$. Two parameters ${\hat{\theta }}^{i,j},{\hat{\theta }}^{i,j+1},\left(i,j\right)\in \left\{1,p_{1}\right\}\times \left\{1,2,\cdots p_{2}-1\right\}$ take part in the interpolation, *i.e.*,

$$\Delta W_{c,T}\left(\theta^{i,j},\upsilon \right)=\frac{n_{e}^{j\;+\;1}-n_{e}}{n_{e}^{j\;+\;1}-n_{e}^{j}}{\hat{\theta }}^{i,j}+\frac{n_{e}-n_{e}^{j}}{n_{e}^{j\;+\;1}-n_{e}^{j}}{\hat{\theta }}^{i,j+1}$$

Case 4: $\left(k,l\right)\in \left\{1,2,\cdots p_{1}-1\right\}\times \left\{1,2,\cdots p_{2}-1\right\}$. Four parameters ${\hat{\theta }}^{i,j}$, ${\hat{\theta }}^{i+1,j}$, ${\hat{\theta }}^{i,j+1}$, ${\hat{\theta }}^{i+1,j+1}$, $\left(i,j\right)\in \left\{1,2,\cdots p_{1}-1\right\}\times \left\{1,2,\cdots p_{2}-1\right\}$ take part in the interpolation, *i.e.*,

$$\begin{array}{cc} \Delta W_{c,T}\left({\hat{\theta }}^{i,j},\upsilon \right) & =\frac{n_{e}^{j\;+\;1}-n_{e}}{n_{e}^{j\;+\;1}-n_{e}^{j}}\frac{u_{\delta }^{i+1}-u_{\delta }}{u_{\delta }^{i+1}-u_{\delta }^{i}}{\hat{\theta }}^{i,j}+\frac{n_{e}^{j\;+\;1}-n_{e}}{n_{e}^{j\;+\;1}-n_{e}^{j}}\frac{u_{\delta }-u_{\delta }^{i}}{u_{\delta }^{i+1}-u_{\delta }^{i}}{\hat{\theta }}^{i+1,j}\\ & +\frac{n_{e}-n_{e}^{j}}{n_{e}^{j\;+\;1}-n_{e}^{j}}\frac{u_{\delta }^{i+1}-u_{\delta }}{u_{\delta }^{i+1}-u_{\delta }^{i}}{\hat{\theta }}^{i,j+1}+\frac{n_{e}-n_{e}^{j}}{n_{e}^{j\;+\;1}-n_{e}^{j}}\frac{u_{\delta }-u_{\delta }^{i}}{u_{\delta }^{i+1}-u_{\delta }^{i}}{\hat{\theta }}^{i+1,j+1} \end{array}$$

In order to expediently discuss the convergence of the parameter estimate ${\hat{\theta }}^{i,j}$ corresponding to different regions $R_{u_{\delta }}^{k}\times R_{n_{e}}^{l}$, a local regression vector $\Psi_{l}\left(\upsilon \right)$ is defined based on the above four classifications of the region partition as follow:

(34) $$\Psi_{l}\left(\upsilon \right)=\left\{\begin{array}{cc} 1 & \mathrm{if}\;\upsilon \in \left(R_{u_{\delta }}^{0}\cup R_{u_{\delta }}^{p_{1}}\right)\times \left(R_{n_{e}}^{0}\cup R_{n_{e}}^{p_{2}}\right)\\ \left(\begin{array}{cc} 1-\eta_{1} & \eta_{1} \end{array}\right) & \mathrm{if}\;\upsilon \in R_{u_{\delta }}^{i}\times \left(R_{n_{e}}^{0}\cup R_{n_{e}}^{p_{2}}\right)\\ \left(\begin{array}{cc} 1-\eta_{2} & \eta_{2} \end{array}\right) & \mathrm{if}\;\upsilon \in \left(R_{u_{\delta }}^{0}\cup R_{u_{\delta }}^{p_{1}}\right)\times R_{n_{e}}^{j}\\ {\left(\begin{array}{c} \left(1-\eta_{1}\right)\left(1-\eta_{2}\right)\\ \eta_{1}\left(1-\eta_{2}\right)\\ \left(1-\eta_{1}\right)\eta_{2}\\ \eta_{1}\eta_{2} \end{array}\right)}^{T} & \mathrm{if}\;\upsilon \in R_{u_{\delta }}^{i}\times R_{n_{e}}^{j} \end{array}\right.$$

where:

(35) $$\begin{array}{c} \eta_{1}=\frac{u_{\delta }-u_{\delta }^{i}}{u_{\delta }^{i+1}-u_{\delta }^{i}},\eta_{2}=\frac{n_{e}-n_{e}^{j}}{n_{e}^{j+1}-n_{e}^{j}}\\ i=1,2,\cdots p_{1}-1;j=1,2,\cdots p_{2}-1 \end{array}$$

When $\upsilon \in R_{u_{\delta }}^{k}\times R_{n_{e}}^{l}$, regression vector $\Psi \left(\upsilon \right)$ in Equation (26) can be replaced by local regression vector $\Psi_{l}\left(\upsilon \right)$; then, observer Equation (26) can be replaced by:

(36) $$\begin{array}{cc} \dot{\hat{x}} & =A\left(\rho \right)\hat{x}+E+L\left(y_{m}-C\left(\rho \right)\hat{x}-G\Psi_{l}\left(\upsilon \right){\hat{\theta }}_{l}^{i,j}\right)\\ {\dot{\hat{\theta }}}_{l}^{i,j} & =\Gamma_{l}\Psi_{l}{\left(\upsilon \right)}^{T}\left(y_{m2}-C_{2}\left(\rho \right)\hat{x}-\Psi_{l}\left(\upsilon \right){\hat{\theta }}_{l}^{i,j}\right) \end{array}$$

where ${\hat{\theta }}_{l}^{i,j}$ is the local parameter estimate of appropriate size and $\Gamma_{l}$ is a local positive definite diagonal matrix of appropriate size.

According to Theorem 1, the local parameter estimate ${\hat{\theta }}_{l}^{i,j}$ is convergent if local regression vector $\Psi_{l}\left(\upsilon \right)$ is persistently exciting. Meanwhile, the parameter estimate $\hat{\theta }$ is also convergent if the trajectory of the map input *υ* passes through all of the interpolation regions $R_{u_{\delta }}^{k}\times R_{u_{2}}^{l}$.

There are heavy matrices calculated in real time for the covariance matrix equation of EKF in [9,10,11], preventing it from being implemented in commercial electronic control units (ECUs) for map adaptation. Nevertheless, the computational burden of the proposed observer Equation (26) without the additional matrix equation is lower. Moreover, the number of parameter estimates $\hat{\theta }$ updated in Equation (26) is no more than four at any time; then, the computational load can be further reduced by stopping estimating ${\hat{\theta }}^{i,j}$ corresponding to $\upsilon \notin R_{u_{\delta }}^{k}\times R_{n_{e}}^{l}$.

**Remark 3.**  *For the area S where the trajectory of the map input υ does not move, the parameters ${\hat{\theta }}^{i,j}$ corresponding to the interpolation region belonging to S cannot be estimated by observer Equation (26). In order to get the map parameters corresponding to S, an extrapolation model can be taken as follows:*

(37) $$\begin{array}{c} \Delta W_{c,e}\left(u_{\delta },n_{e}\right)=a_{2}u_{\delta }^{2}\;+\;a_{1}u_{\delta }+b_{2}n_{e}^{2}+b_{1}n_{e}+c_{2}u_{\delta }n_{e}+c_{1} \end{array}$$

*
where $a_{2},a_{1},b_{2},b_{1},c_{2},c_{1}$ are polynomial parameters. Based on the data from the estimated map parameters, extrapolation model Equation (37) can be fitted by polynomial fitting approach, and then map parameters corresponding to S can be obtained*.

## 4. Simulation Results

**Figure 2:**
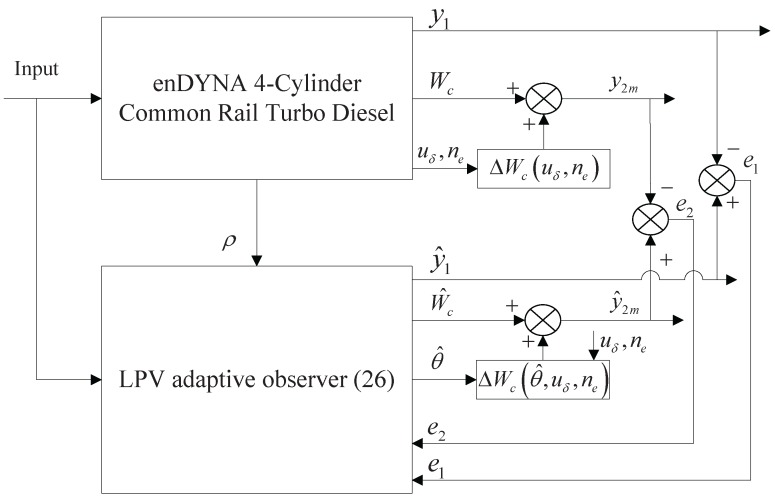
Schematic diagram of the adaptive observer.

In this section, the simulation study of 2D map estimation is presented in the environment of a 1.9 L four-cylinder common rail turbo diesel engine of enDYNA, in which the ETC and FTP75 are used as test conditions, respectively. The observer architecture is illustrated in Figure 2, where $\Delta W_{c}\left(u_{\delta },n_{e}\right)$ is the additive reference error as the true measurement error from enDYNA.

Bounds on the parameter vector *ρ* are presented in Table 1. When the inequality Equations (28) and (33) are solved with $\varepsilon_{1}=0.25$ and $\varepsilon_{2}=0.11$, the gain matrix *L* can be given by:

(38) $$\begin{array}{c} L=\left(\begin{array}{ccc} \text{1635.83} & -\text{124.95} & \text{6.52}\times {\text{10}}^{-7}\\ -\text{16,063.4} & \text{3135.43} & \text{1.67}\times {\text{10}}^{-7}\\ \text{4.2} & \text{29.7} & \text{4.55}\times {\text{10}}^{-20} \end{array}\right) \end{array}$$

**Figure 3:**
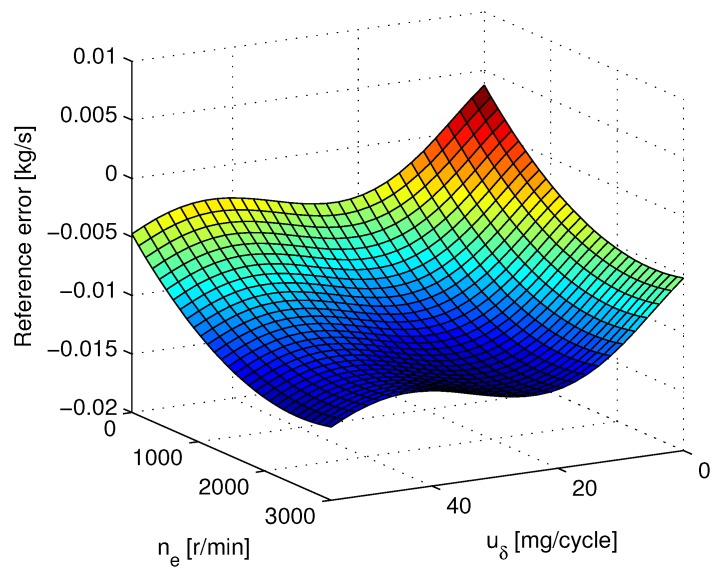
Reference error $\Delta W_{c}\left(u_{\delta },n_{e}\right)$ used as the mass air flow (MAF) sensor error in enDYNA.

The initial values of observer Equation (26) used in the simulation are $\hat{x}\left(0\right)={\left[\begin{array}{ccc} 9.8\times 10^{5} & 9.8\times 10^{5} & 0 \end{array}\right]}^{T},\hat{\theta }\left(0\right)=0$, and the parameter gain is Γ=200I. Here, the reference error $\Delta W_{c}\left(u_{\delta },n_{e}\right)$ assumed as MAF sensor measurement error is depicted in Figure 3, which is superimposed on the signal $W_{c}$ in enDYNA as the measured value $y_{m2}$ in the simulation.

### 4.1. 2D Map Estimation under ETC

**Figure 4:**
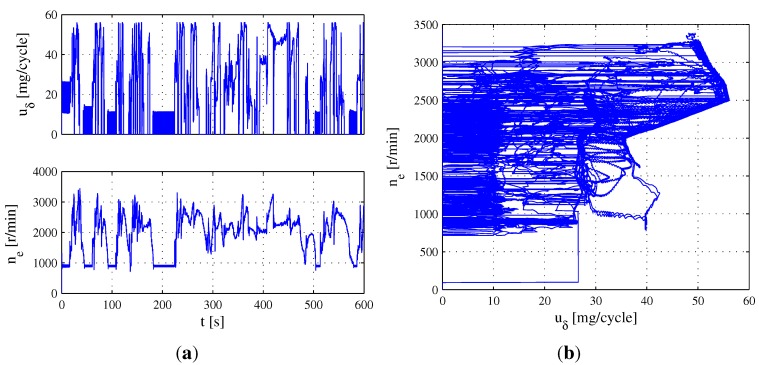
Evolution of operating point $\upsilon =\left(u_{\delta },n_{e}\right)$ during ETC part one. (**a**) Evolution of $u_{\delta }$ and $n_{e}$. (**b**) Trajectory of operating point $\upsilon =\left(u_{\delta },n_{e}\right)$.

There are three parts of the ETC representing three different driving conditions, including urban, rural and motorway driving. Due to the engine speed range from ETC Part 1 covering the other two parts, ETC Part 1 is employed as the test condition in this section. Accordingly, the fuel mass injection quantity $u_{\delta }$ and engine speed $n_{e}$ from ETC Part 1 are plotted in Figure 4a, and the trajectory of the operating point $\upsilon =\left(u_{\delta },n_{e}\right)$ is depicted in Figure 4b, in which the trajectory does not move in area $S=\left[\left(0,56\right)\times \left(0,800\right)\right]\cup \left[\left(40,56\right)\times \left(0,2000\right)\right]$. According to the range $u_{\delta }\in \left[0,56\right]$ and $n_{e}\in \left[0,3100\right]$ from Figure 4a, an average partition can be respectively given as [0:4:56] and [0:250:3000].

**Figure 5:**
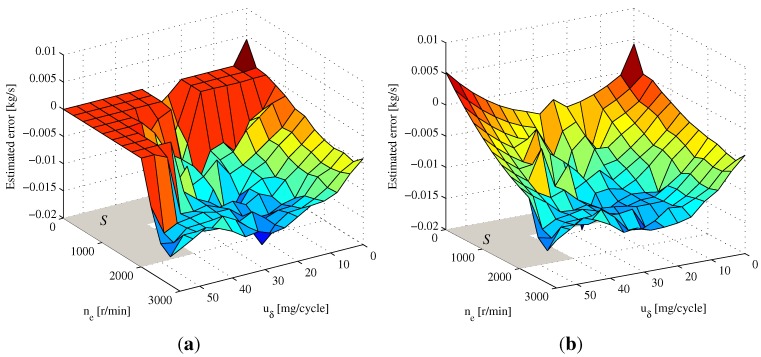
The estimated 2D map for the MAF sensor error. (**a**) Estimation results of the 2D map under ETC Part 1; (**b**) Extrapolation results based on the estimated 2D map.

The estimation results of the MAF sensor error using observer Equation (26) under ETC Part 1 are shown in Figure 5a, in which the map parameters have been estimated, except area *S*. According to Remark 3, the map parameters corresponding to area *S* can be obtained from the extrapolation model Equation (37). Based on the estimated map parameters from Figure 5a, the polynomial parameters in Equation (37) can be fitted as follows:

(39) $$\begin{array}{c} a_{2}=9.12\times 1{\text{0}}^{-6},a_{1}=-4.39\times 1{\text{0}}^{-4},b_{2}=1.30\times 1{\text{0}}^{-9}\\ b_{1}=-5.74\times 1{\text{0}}^{-6},c_{2}=-5.37\times 1{\text{0}}^{-8},c_{1}=0.0012887 \end{array}$$

The parameters corresponding to area *S* obtained from Equation (37) are presented in Figure 5b, which can roughly reflect the trend of the map.

**Figure 6:**
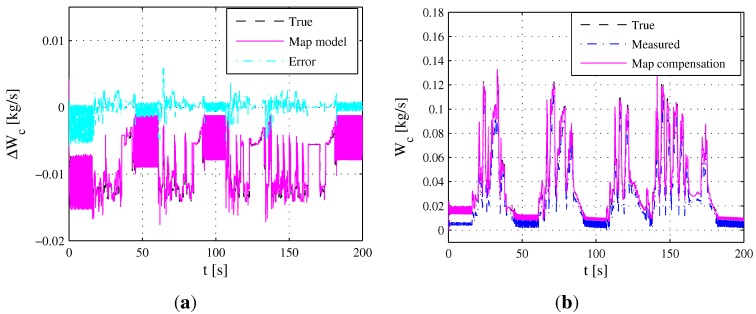
2D map compensation during the ETC segment. (**a**) Simulation results between reference error $\Delta W_{c}\left(u_{\delta },n_{e}\right)$ and the estimated 2D map; (**b**) Simulation results of true mass air flow $y_{2}$, measured mass air flow $y_{m2}$ and the map compensation.

In order to evaluate the accuracy of the estimated 2D map shown in Figure 5a, the comparison between the reference error $\Delta W_{c}\left(u_{\delta },n_{e}\right)$ and the estimated 2D map during the ETC segment is presented in Figure 6a. Accordingly, the true mass air flow $y_{2}$, measured mass air flow $y_{m2}$ and map compensation are shown in Figure 6b. The mean relative error between reference error $\Delta W_{c}\left(u_{\delta },n_{e}\right)$ and estimated 2D map is 10.41%, which demonstrates that the measured output $y_{m2}$ of the MAF sensor after map correction can approximate the true value of $W_{c}$ acceptably.

### 4.2. 2D Map Estimation under FTP75

**Figure 7:**
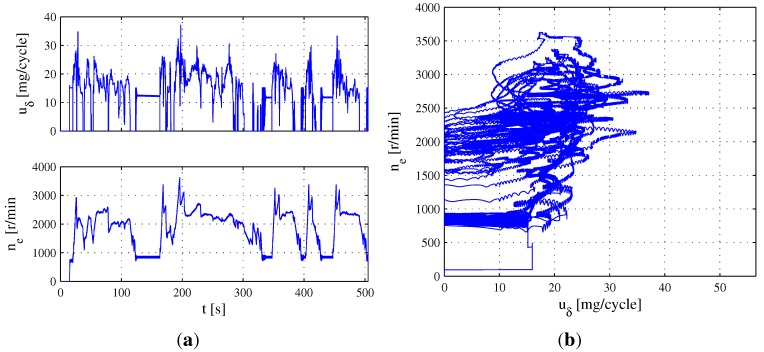
Evolution of operating point $\upsilon =\left(u_{\delta },n_{e}\right)$ FTP75 cold start transient phase. (**a**) Evolution of $u_{\delta }$ and $n_{e}$; (**b**) Trajectory of operating point $\upsilon =\left(u_{\delta },n_{e}\right)$.

In order to verify the effectiveness of the proposed method under different conditions, the cold start transient phase of the FTP75 is used in this section. Accordingly, $u_{\delta }$ and $n_{e}$ are plotted in Figure 7a, and the trajectory of *υ* is depicted in Figure 7b, in which the trajectory does not move in area $S=\left[\left(0,56\right)\times \left(0,650\right)\right]\cup \left[\left(25,37\right)\times \left(0,2000\right)\right]\cup \left[\left(37,56\right)\times \left(0,3500\right)\right]$.

**Figure 8:**
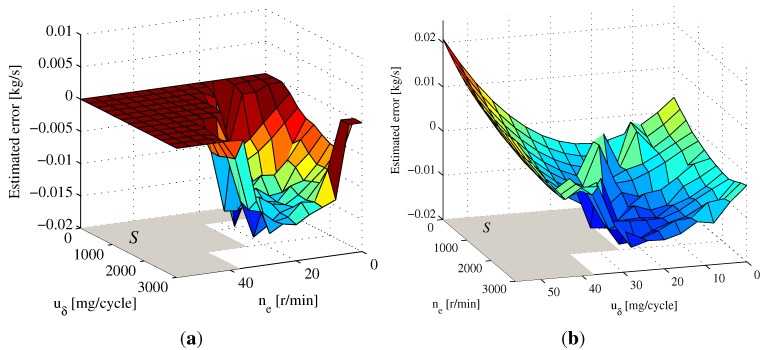
The estimated 2D map for the MAF sensor error. (**a**) Estimation results of the 2D map under FTP75 cold start transient phase; (**b**) Extrapolation results based on the estimated 2D map.

The estimation results of the MAF sensor error under the cold start transient phase of the FTP75 are shown in Figure 8a, and the polynomial parameters in Equation (37) are fitted as follows:

(40) $$\begin{array}{c} a_{2}=2.10\times 1{\text{0}}^{-5},a_{1}=-8.62\times 1{\text{0}}^{-4},b_{2}=1.46\times 1{\text{0}}^{-9}\\ b_{1}=-6.40\times 1{\text{0}}^{-6},c_{2}=-1.84\times 1{\text{0}}^{-8},c_{1}=0.0032316 \end{array}$$

**Figure 9:**
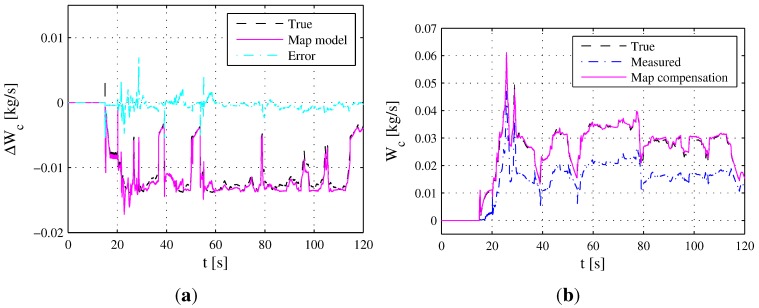
2D map compensation during the FTP75 segment. (**a**) Simulation results between reference error $\Delta W_{c}\left(u_{\delta },n_{e}\right)$ and the estimated 2D map; (**b**) Simulation results of true mass air flow $y_{2}$, measured mass air flow $y_{m2}$ and the map compensation.

The map added the parameters corresponding to area *S* are shown in Figure 8b, which can also roughly reflect the trend of the map. Under the FTP75 segment, the comparison between the reference error $\Delta W_{c}\left(u_{\delta },n_{e}\right)$ and the estimated 2D map from Figure 8a is shown in Figure 9a. Accordingly, the MAF sensor measured value $y_{m2}$ using map compensation is presented in Figure 9b. The mean relative error between reference error $\Delta W_{c}\left(u_{\delta },n_{e}\right)$ and the estimated 2D map is 5.28%, demonstrating that the measured output $y_{m2}$ after map correction can approximate the true value of $W_{c}$ acceptably.

## 5. Conclusions

A method for updating and storing sensor bias from different operating points is developed and investigated. This method achieves simultaneous estimation of model states and map parameters and applies to updating the MAF sensor error 2D map in the engine. The map in the form of a vector-vector dot product is given to conveniently analyze and design the parameter estimation method. An LPV adaptive observer to estimate map parameters is designed, which has the advantage of a simple structure and low computational load. Under ETC Part 1 and the cold start transient phase of the FTP75, the effectiveness of the presented algorithm is verified and validated in the engine software enDYNA. The results demonstrate that the proposed method can estimate the MAF sensor error acceptably.

## Nomenclature

$p_{amb}$	ambient pressure (Pa)	$p_{im}$	intake manifold pressure (Pa)
$p_{em}$	exhaust manifold pressure (Pa)	$\omega_{t}$	turbine speed (rad/s)
$n_{e}$	engine speed (rpm)	$W_{c}$	compressor mass air flow (kg/s)
$W_{egr}$	EGR mass flow (kg/s)	$W_{ei}$	cylinder mass flow (kg/s)
$W_{f}$	fuel rate injected to cylinder (kg/s)	$W_{t}$	turbine mass flow (kg/s)
$P_{c}$	compressor power (W)	$P_{t}$	turbine power (W)
$\Phi_{c}$	volumetric flow coefficient	$\Psi_{egr}$	energy transfer coefficient
$\eta_{vol}$	volumetric efficiency	$\eta_{tm}$	turbine efficiency
$\eta_{m}$	turbocharger mechanical efficiency	$\eta_{c}$	compressor efficiency
$T_{amb}$	ambient temperature (K)	$T_{im}$	intake manifold temperature (K)
$T_{em}$	exhaust manifold temperature (K)	$V_{im}$	intake manifold volume (m${}^{3}$)
$V_{em}$	exhaust manifold volume (m${}^{3}$)	$V_{d}$	displaced volume (m${}^{3}$)
$R_{a}$	air gas constant (J/(kg·K))	$R_{e}$	exhaust gas constant (J/(kg·K))
$R_{c}$	compressor blade radius (m)	$\gamma_{a}$	air specific heat capacity ratio
$\gamma_{e}$	exhaust specific heat capacity ratio	$\Pi_{c}$	compressor pressure quotient
$\Pi_{t}$	turbine pressure quotient	$J_{t}$	turbine inertia (kg·m${}^{2}$)
$n_{cyl}$	number of cylinders	$A_{egr}$	EGR valve effective area (m${}^{2}$)
$A_{vgtmax}$	VGT nozzle maximum effective area (m${}^{2}$)	$f_{\Pi_{t}}$	choking function
$f_{vgt}$	effective area ratio function	$u_{egr}$	EGR valve opening percentage (%)
$u_{vgt}$	VGT vane opening percentage (%)	$u_{\delta }$	injected amount of fuel (mg/cycle)
$c_{pa}$	air specific heat capacity at constant pressure (J/(kg·K))
$c_{pe}$	exhaust specific heat capacity at constant pressure (J/(kg·K))
